# Prenatal air pollution and children’s autism traits score: Examination of joint associations with maternal intake of vitamin D, methyl donors, and polyunsaturated fatty acids using mixture methods

**DOI:** 10.1097/EE9.0000000000000316

**Published:** 2024-06-21

**Authors:** Megan G. Bragg, Irena Gorski-Steiner, Ashley Song, Jorge E. Chavarro, Jaime E. Hart, Loni P. Tabb, Marc G. Weisskopf, Heather Volk, Kristen Lyall

**Affiliations:** a AJ Drexel Autism Institute, Drexel University, Philadelphia, Pennsylvania; b Department of Mental Health, Johns Hopkins Bloomberg School of Public Health, Johns Hopkins University, Baltimore, Maryland; c Department of Nutrition, Harvard T.H. Chan School of Public Health, Boston, Massachusetts; d Channing Division of Network Medicine, Department of Medicine, Brigham and Women’s Hospital and Harvard Medical School, Boston, Massachusetts; e Department of Environmental Health, Harvard T.H. Chan School of Public Health, Boston, Massachusetts; f Department of Epidemiology and Biostatistics, Dornsife School of Public Health, Drexel University, Philadelphia, Pennsylvania; Pennsylvania State University; Emory University; UC Davis; Kaiser Permanente

**Keywords:** Neurodevelopment, Statistical methods, Maternal diet, Environmental exposures

## Abstract

**Background:**

Maternal nutrient intake may moderate associations between environmental exposures and children’s neurodevelopmental outcomes, but few studies have assessed joint effects. We aimed to evaluate whether prenatal nutrient intake influences the association between air pollutants and autism-related trait scores.

**Methods:**

We included 126 participants from the EARLI (Early Autism Risk Longitudinal Investigation, 2009–2012) cohort, which followed US pregnant mothers who previously had a child with autism. Bayesian kernel machine regression and traditional regression models were used to examine joint associations of prenatal nutrient intake (vitamins D, B12, and B6; folate, choline, and betaine; and total omega 3 and 6 polyunsaturated fatty acids, reported via food frequency questionnaire), air pollutant exposure (particulate matter <2.5 μm [PM_2.5_], nitrogen dioxide [NO_2_], and ozone [O_3_], estimated at the address level), and children’s autism-related traits (measured by the Social Responsiveness Scale [SRS] at 36 months).

**Results:**

Most participants had nutrient intakes and air pollutant exposures that met US standards. Bayesian kernel machine regression mixture models and traditional regression models provided little evidence of individual or joint associations of nutrients and air pollutants with SRS scores or of an association between the overall mixture and SRS scores.

**Conclusion:**

In this cohort with a high familial likelihood of autism, we did not observe evidence of joint associations between air pollution exposures and nutrient intake with autism-related traits. Future work should examine the use of these methods in larger, more diverse samples, as our results may have been influenced by familial liability and/or relatively high nutrient intakes and low air pollutant exposures.

**What this study adds**

Maternal diet during pregnancy may moderate associations of air pollutants with children’s neurodevelopmental outcomes, including autism. Previous studies have examined nutrients and air pollutants as independent predictors of autism outcomes or have examined only individual nutrients as potential moderators. We used mixture models to examine the joint associations of a suite of nutrients and air pollutants with children’s autism-related traits. Although we found little evidence for individual or joint associations of exposures with autism-related traits in a familial autism cohort, this study highlights the ability of mixture models to examine complex associations of nutrients and environmental exposures with children’s neurodevelopment.

## Introduction

Autism is a neurodevelopmental diagnosis characterized by challenges with social communication and the presence of restricted repetitive behaviors. In 2020, 1 in 36 US children aged 8 years old was diagnosed with autism.^1^ Evidence suggests that broader traits related to autism and social communication are distributed continuously among the general population and suggests the utility of quantitative trait measures in etiologic investigations of autism.^2,3^ While the etiology of autism is unclear, both genetic and environmental factors have been associated with autism diagnosis and quantitative traits related to the autism phenotype.^4,5^ For environmental exposures, evidence suggests the perinatal period represents a sensitive window for influences on autism development.^6,7^

One such environmental exposure with evidence for relationships with autism is air pollution. Exposure to criteria air pollutants, including fine particulate matter less than 2.5 micrometers (PM_2.5_), nitrogen dioxide (NO_2_), and ozone (O_3_), is ubiquitous and is influenced by traffic and industrial emissions.^8^ These exposures have been consistently associated with a range of neurodevelopmental and health outcomes, including autism diagnosis and quantitative traits,^9,10^ even at levels below US regulatory standards.^11^ It is hypothesized that air pollutants influence fetal brain development via increased oxidative stress, neuroinflammation, and altered gene methylation.^12–14^

Prenatal diet is another environmental factor that has been associated with children’s autism development and neurodevelopment more broadly. Intake of folate, vitamin D, and polyunsaturated fatty acids (PUFAs) in particular has been inversely associated with autism and related traits.^15^ In addition to independent effects, nutrients may interact with environmental exposures through common biological pathways to either attenuate (when present in sufficient amounts) or exacerbate (when levels are suboptimal) neurodevelopmental effects.^16,17^ The interaction between prenatal diet and environmental toxins in relationship to neurodevelopmental outcomes was recently reviewed.^18^ There is suggestive evidence that prenatal nutrient intake may moderate the association of air pollutants and other environmental exposures with autism-related outcomes. For example, maternal intake of folic acid greater than 800 mcg in the first month of pregnancy attenuated the association between prenatal air pollutants and autism diagnosis among 760 children in a case–control study in California.^19^ Given that pregnant individuals may be better able to change their diet than their environmental air pollution exposure, this represents a potential target for public health messaging and interventions.

Although promising, previous work has been limited in the number of nutrients examined. For example, no studies have examined the moderating role of vitamin D or PUFAs on the association of air pollutants and autism, although a few studies have examined other environmental exposures and neurodevelopmental outcomes.^20–23^ In addition, although folate is hypothesized to act via changes to methylation pathways,^24^ other nutrients on the same pathways (vitamin B12, vitamin B6, choline, and betaine) have been less studied. Perhaps one reason the number of nutrients has been limited is the need for statistical techniques that can accommodate several potentially multicollinear exposures. Nutrient intakes are often highly correlated due to the combined consumption of foods and may also have biological interactions. Mixture methods such as Bayesian kernel machine regression (BKMR) are designed to assess mixture effects by reducing concerns about multicollinearity or multiple testing.^25^ Previously, we used BKMR to examine the association of multiple dietary factors with a quantitative autism traits score;^26^ however, to our knowledge, no studies have examined the association of both dietary factors and air pollutants with autism outcomes within a single mixture model.

The goal of this analysis was to evaluate the joint associations of prenatal air pollutant exposure and nutrient intake (vitamin D, folate, vitamin B12, vitamin B6, choline, betaine, total omega 3, and total omega 6 PUFAs) with autism traits scores using BKMR. As a comparison to test bivariate interactions and effect modification, we also performed traditional regression models of air pollutant exposure and autism traits score, stratified by nutrient intake. We hypothesized that air pollutants would be positively associated with autism traits and that this relationship would be attenuated with higher nutrient intake.

## Methods

### Study design and participants

This study uses data from the Early Autism Risk Longitudinal Investigation (EARLI; 2009-2012), which followed pregnant participants who had a previous child with autism.^27^ This cohort has an increased likelihood of autism given the high sibling recurrence risk.^28^ Eligibility criteria for EARLI included the ability to communicate in English or Spanish, being 18 years or older, living within 2 hours of a study site, and being less than 29 weeks pregnant. EARLI study sites were in four US locations (Philadelphia, PA [Drexel University], Baltimore, MD [Johns Hopkins University], San Francisco, CA [Kaiser Permanente Northern California], and Sacramento, CA [University of California, Davis]). Participants visited their study sites once per trimester and at birth to complete questionnaires and collect biological and environmental samples. Ethics approval for EARLI was obtained from the Drexel University Institutional Review Board, and all maternal participants provided written consent. To be selected for the current study, mother–child pairs needed to have a geocoded address during pregnancy, prenatal nutrient intake data, and measurement of children’s autism traits at 36 months of age. For sets of twins, one child was randomly excluded from the analysis.

### Autism-related outcomes

Two autism-related outcomes were measured in EARLI: autism diagnosis and quantitative autism-related traits, as measured by the Social Responsiveness Scale (SRS),^29^ at 36 months of age. Due to the small sample size and limited number of autism cases, as well as the established use of continuous outcomes in BKMR models,^25,26^ SRS scores were used as the primary outcome in this analysis.

SRS scores were determined based on a 65-item caregiver report (SRS preschool version) to assess quantitative measures of autistic phenotype.^29^ Raw scores were generated by summing individual item scores, with higher SRS raw scores indicating higher expression of autism-related traits (i.e., more deficits in social communications).^2^ The SRS has been validated against gold-standard autism diagnostic tools (*r* > 0.6).^30^

### Air pollution exposure estimation

Prenatal air pollution exposure was estimated at each residential address. Addresses were standardized and geocoded using the TeleAtlas US_Geo_2 database and software (Tele Atlas, Inc., Boston, CA, www.geocoded.com). Ozone exposures were derived using data from the US Environmental Protection Agency’s (EPA) Air Quality System (www.epa.gov/aqs). Weekly air quality data were spatially interpolated based on an inverse distance-squared weighting of data from up to four closest stations located within 50 km of each participant's residence;^31^ if one or more stations were located within 5 km of a residence, then only data from the stations within 5 km were used for interpolation. PM_2.5_ and NO_2_ exposures were derived using data from a spatiotemporal ensemble model that predicts daily concentrations at 1 km^2^ spatial resolution by integrating different machine learning algorithms and utilizing over 100 predictors including satellite measurements, land-use terms, meteorological variables, and chemical transport model predictions.^32^ ArcGIS Pro’s ModelBuilder tool was used to match pregnancy dates and geocoded addresses to the data extracted from this model and calculate daily exposure estimates for each mother–child pair.^33^ Estimates for each pollutant were averaged across pregnancy and by trimester. Air pollutant exposures were compared to current EPA primary annual standards for PM_2.5_ and NO_2_.^8^ The EPA does not have an annual standard for ozone, so no comparison with our data was made.

### Nutrient intake calculation

Participants completed a validated food frequency questionnaire (the National Cancer Institute’s Diet History Questionnaire II^34^ slightly modified for pregnancy) at ~20 weeks gestation, representing the first half of pregnancy, and at the end of pregnancy, representing the second half. Data representing the first half of pregnancy were selected for primary analyses to maximize sample size and to align with previous work highlighting the importance of nutrient intake in the periconceptional and early pregnancy period.^35,36^ The questionnaire asked participants to report their average intake of a list of 124 foods and beverages. Nutrient intake was derived from the Diet History Questionnaire II using standard nutrient composition databases and then adjusted for total energy intake using the nutrient residual energy adjustment method.^37^ A separate questionnaire was administered to capture participants’ use of supplements during each month of pregnancy. Average nutrient intake from supplements was calculated for all nutrients except choline, betaine, and total omega 6 fatty acids, for which information on levels in supplements was unavailable. Data from nationally representative samples suggest that supplemental intake of these nutrients is uncommon among pregnant and lactating individuals.^38^ Total energy-adjusted nutrient intake from diet and, where available, from supplements was calculated. A suite of high-priority nutrients was identified based on previous associations with autism outcomes:^15^ vitamin D, folate, and other nutrients involved in methylation pathways (vitamin B12, vitamin B6, choline, and betaine), and total omega 3 and total omega 6 PUFAs. When available, the percentage of participants with intake above the US National Academies of Science and Medicine Recommended Dietary Allowance (RDA), Adequate Intake (AI), and Upper Level (UL) during pregnancy was calculated for each nutrient.

### Statistical analysis

Characteristics of those included in this analysis versus the overall EARLI sample were compared. We then examined univariate distributions and bivariate relations among study variables to check for collinearity and confounding using variance inflation factors, scatterplots, box plots, and Spearman correlations. All models were run unadjusted and adjusted for covariates.

We employed BKMR to examine associations of exposures with SRS score. BKMR captures high dimensional, complex exposure–response associations and can investigate interactions among multiple exposures.^25^ Analyses were performed using the R package *bkmr* (R Core Team, Vienna, Austria)*.* Additionally, the R package *bkmrhat* was used to assess model fit. The number of iterations was increased until convergence was achieved.^25^ Air pollutants and nutrients are hypothesized to be positively and negatively associated with autism trait scores, respectively. For clearer interpretation of mixture effects, air pollutants were reverse coded (all observations multiplied by −1) prior to inclusion in BKMR models so that a positive association with SRS would indicate higher SRS scores with lower air pollution exposure. Given the variability in the distributions of air pollutant and nutrient exposures, all exposures were standardized using a z-score. BKMR allows for exposures to be individually examined or assigned to groups based on correlation or shared hypothetical pathways. We ran both individualized and grouped analyses, assigning separate groups for air pollutants, nutrients on the methylation pathway (folate, vitamin B12, vitamin B6, choline, and betaine), fatty acids (total omega 3s, total omega 6s), and vitamin D. Given its Bayesian nature, *P* values are generally not utilized in BKMR and power calculations are not directly applicable.^39^ However, simulation studies and prior literature have demonstrated the ability of BKMR and similar mixture models to detect modest associations in sample sizes of 100–200.^25,40,41^ Figures are presented to illustrate the associations of the full mixture, individual exposures, and bivariate exposures with outcomes. Additionally, posterior inclusion probabilities (PIPs) were calculated, which describe the relative strength of the associations of individual and grouped exposures with the outcome.

To compare with previous work and for direct examination of effect modification with numerical estimates, we also performed traditional regression models. Each nutrient and air pollutant was examined for its independent association with the SRS score using quantile regression at the 50th percentile to account for the skewed outcome distribution. Then, nutrients were individually tested as moderators of the relationship between air pollutants and SRS score using stratified models and interaction terms. For stratified models, potential stratification points were examined based on recommended nutrient intake level (i.e., RDA, AI, or UL). For nutrients without an established recommended intake level during pregnancy, or for nutrients where these stratification points provided little variability in our sample, stratification at the median was performed. Separately, interaction terms of nutrients by air pollutants were tested in regression models of SRS scores. Given stratified samples of n ~60, we were powered to detect effect sizes similar to those seen in prior literature (~3.9 points of the raw SRS score, using two-sided tests, alpha 0.05, and 80% power).^42,43^

### Covariates

Covariates considered in adjusted models were selected based on a priori associations: EARLI study site, child sex (male/female as designated by parent), maternal race and ethnicity (non-Hispanic White, Hispanic, other race/ethnicities as self-reported by mother), maternal age, and an index of individual socioeconomic deprivation derived from self-reported indicators for maternal and paternal education, household income, marital status, and home ownership.

In sensitivity analyses, we also evaluated secondary covariates, which included: child birth year and season, gestational age, and breastfeeding status; maternal prenatal supplement use in the first month of pregnancy, interpregnancy interval between the most recent pregnancy and the current, antidepressant use, prepregnancy body mass index, gestational weight gain, parity, and Alternative Healthy Eating Index score for pregnancy (a measure of diet quality).^44^ Additionally, we examined census tract-based socioeconomic status using the index of concentration at the extremes for community-level race/ethnicity and income.^45^ We did not evaluate physical activity or smoking due to heavy missingness in the cohort (>25%). For all other covariates, missingness was ≤15% so imputation of medians and modes was performed. Similar results were observed in the traditional regression models when missing values were imputed using multiple imputations by chained equations.

### Exploratory analyses

Because the timing of prenatal air pollutant and nutrient exposures may influence neurodevelopmental outcomes including autism development,^6,15^ we performed exploratory analyses to determine whether our findings differed by time point in pregnancy. First, we evaluated associations with trimester-specific averages of air pollutant exposure, with all trimester values in the same model for mutual adjustment. Second, we examined associations when using nutrient intake from the second half of pregnancy. These analyses were only performed in the primary BKMR models. Finally, we also conducted parallel nutrient stratified and BKMR probit analyses of autism diagnosis; given the small number of autism cases in our sample, these were considered for broad comparison to primary analyses only.

For all analyses, *P* ≤0.05 was considered statistically significant.

## Results

### Characteristics of the study population

**Figure 1.**
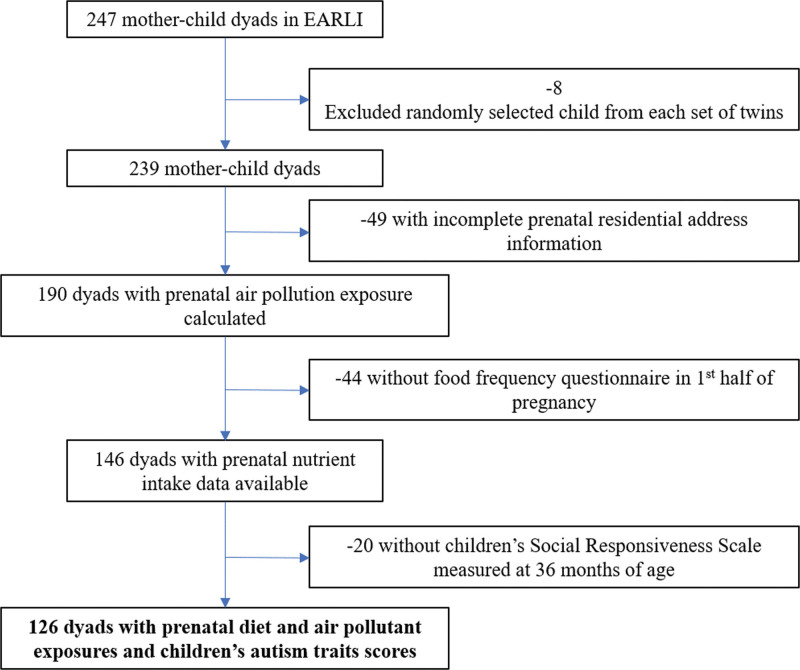
Selection flow diagram for EARLI participants included in this analysis of nutrients, air pollutants, and autism outcomes. EARLI – Early Autism Risk Longitudinal Investigation

A total of 126 mother–child pairs were included in this analysis (Figure 1). Mothers were primarily non-Hispanic White with advanced education (Table 1). Almost 60% of mothers reported the use of prenatal vitamins during the first month of pregnancy; 93.7% used prenatal vitamins ever in their pregnancy. At 36 months of age, median raw total SRS scores was 29.5 (interquartile range: 18.0–42.0), and 29 (23.0%) of children were diagnosed with autism using gold-standard measures. Though our analytic sample was broadly comparable to the full EARLI study overall, included participants were more likely to be non-Hispanic White and have an annual household income >$50k (eTable 1; http://links.lww.com/EE/A285).

**Table 1.** Characteristics of EARLI participants with measured nutrient intake, air pollution exposure, and autism outcomes (N = 126)

	Median (IQR) or n (%)
Child characteristics	
Female, n (%)	55 (43.7)
SRS total raw score	29.5 (18.0–42.0)
Autism diagnosis, n (%)^a^	29 (23.0)
Maternal and familial characteristics	
Maternal age (year)	34.0 (31.0–37.0)
Maternal race/ethnicity^b^, n (%)	
Non-Hispanic White	77 (61.1)
Hispanic	24 (19.0)
Other	25 (19.8)
Parity (number of live children prior to study pregnancy), n (%)	
1	61 (48.4)
2	44 (34.9)
3 or more	20 (15.9)
Missing	1 (0.8)
Used prenatal vitamin/supplement during first month of pregnancy, n (%)	
Yes	72 (57.1)
No	53 (42.1)
Missing	1 (0.8)
Individual socioeconomic deprivation index	0.3 (0.1–0.6)
Maternal education, n (%)	
High school or less	16 (12.7)
Some college or more	109 (86.5)
Missing	1 (0.8)
Paternal education, n (%)	
High school or less	26 (20.6)
Some college or more	96 (76.2)
Missing	4 (3.2)
Household income, n (%)	
<$50K	25 (19.8)
$50–100K	46 (36.5)
$100K+	51 (40.4)
Missing	4 (3.2)
Marital status, n (%)	
Married	22 (17.5)
All other situations	103 (81.7)
Missing	1 (0.8)
Homeownership, n (%)	
Owned with a mortgage	76 (60.3)
Owned free and clear	4 (3.2)
Rented for cash	44 (34.9)
Occupied without rent	1 (0.8)
Missing	1 (0.8)
EARLI site, n (%)	
Drexel	33 (26.2)
Johns Hopkins	30 (23.8)
Kaiser Permanente	46 (36.5)
UC Davis	17 (13.5)

a Two children did not have information available regarding autism diagnosis.

b Race and ethnicity were combined and collapsed due to small n’s <5 in cells.

IQR indicates interquartile range.

### Nutrient and air pollutant exposures

Maternal nutrient intake from diet and supplements during the first half of pregnancy is reported in Table 2. About half of mothers reported vitamin D intake above the RDA, whereas almost all mothers reported intake above the RDA for folic acid, vitamin B12, and vitamin B6. For folic acid, 88.1% of mothers reported intake above the UL. Choline intake was low, with only 7.1% of mothers reporting intake above the AI. The US National Academies of Sciences, Engineering, and Medicine have not established recommended intakes during pregnancy for betaine, total omega 3s, or total omega 6s. However, there are recommendations for alpha-linolenic acid (ALA; an essential omega 3 fatty acid) and linoleic acid (LA; an essential omega 6 fatty acid). Only 4.8% and 25.4% of mothers reported intake above the AI for ALA and LA, respectively.

**Table 2.** Prenatal nutrient intake from diet and supplements and exposure to air pollutants among EARLI participants (N = 126)

	Median (IQR) or n (%)
Nutrients	
Vitamin D (mcg/d)	15.5 (11.2–25.7)
Above RDA (15 mcg)	64 (50.7)
Above UL (100 mcg)	6 (4.8)
Folate (mcg DFE/d)	1835.9 (1346.5–2273.5)
Above RDA (600 mcg DFE)	124 (98.4)
Above UL (1000 mcg DFE)	111 (88.1)
Vitamin B12 (mcg/d)	11.6 (7.9–19.1)
Above RDA (2.6 mcg)	126 (100.0)
Vitamin B6 (mg/d)	4.5 (3.7–12.6)
Above RDA (1.9 mg)	124 (98.4)
Above UL (100 mg)	1 (0.8)
Choline (mg/d)^a^	306.9 (253.6–366.2)
Above AI (450 mg)	9 (7.1)
Above UL (3500 mg)	0 (0.0)
Betaine (mg/d)^a^	95.2 (77.8–109.9)
Total omega 3s (g)	1.2 (1.1–1.4)
Total omega 6s (g)^a^	11.9 (10.6–13.1)
Air pollutants	
PM_2.5_ (µg/m³)	9.9 (8.7–10.7)
Above EPA primary annual standard (12 µg/m³)	9 (7.1)
NO_2_ (ppb)	22.8 (18.6–27.4)
Above EPA primary annual standard (53 ppb)	0 (0.0)
O_3_ (ppb)	25.8 (22.7–29.1)

a Does not include intake from supplements.

DFE indicates dietary folate equivalents; IQR, interquartile range.

Averaged pregnancy air pollutant exposures were below EPA annual standards for PM_2.5_ (12 μg/m^3^) and NO_2_ (53 ppb) for most mothers (Table 2).

Nutrient intakes and air pollutant exposures were weakly to moderately correlated, with Spearman correlations ranging from −0.18 to 0.70 (eTable 2; http://links.lww.com/EE/A285).

### Mixtures models

In covariate-adjusted BKMR models, O_3_ and vitamin B6 had the highest individual PIP values, and air pollutants had the highest group PIP, indicating these exposures had the strongest association with SRS scores (eTable 3; http://links.lww.com/EE/A285).

**Figure 2.**
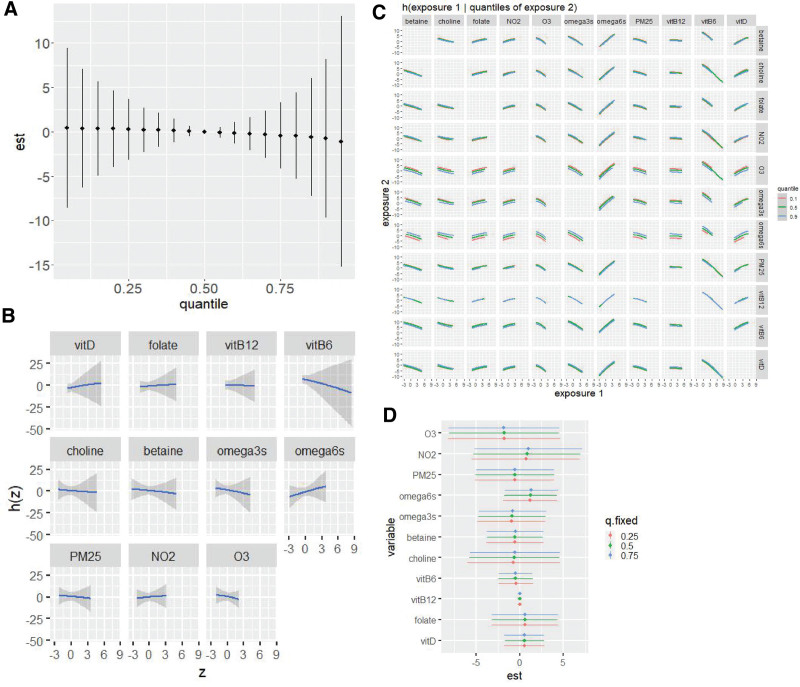
Bayesian kernel machine regression (BKMR) results for the association of prenatal nutrient intake and air pollution exposures with child SRS total raw score in the EARLI study (N = 126). Results are adjusted for study site, maternal age, maternal race and ethnicity, maternal socioeconomic deprivation index, and child sex. Air pollutant values are reverse coded. A, The association of the overall mixture with SRS score, when all exposures are set at the same quantile, compared to when all exposures are at their 50th percentile. B, The independent associations of each exposure with SRS, with all other exposures at their 50th percentile. C, The association of a single exposure with SRS score, when a second exposure is set at varying quantiles, illustrating potential bivariate interactive effects. D, The association of a single exposure with SRS score as it increases from its 25th to 75th percentile while all other exposures are set at specific quantiles. For all plots, covariates are held constant.

There were no statistically significant associations of the overall mixture (Figure 2A) or individual exposures (Figure 2B) with SRS score. There was little evidence for bivariate exposure interactions, as evidenced by a lack of intersecting slopes in Figure 2C. There was also little evidence for interactions of individual exposures with the overall mixture, as illustrated by the similarity of estimates for individual associations at different levels of the mixture (Figure 2D).

These null results remained similar after additional adjustment for covariates (eFigure 1; http://links.lww.com/EE/A285), or use of trimester-specific air pollution exposure (eFigure 2; http://links.lww.com/EE/A285), nutrient intakes during the second half of pregnancy (eFigure 3; http://links.lww.com/EE/A285), or autism diagnosis as an outcome (eFigure 4; http://links.lww.com/EE/A285)

### Traditional regression models

Similar to BKMR results, most exposures were not independently associated with SRS or autism diagnosis in adjusted regression models (eTable 4; http://links.lww.com/EE/A285). When stratified by nutrient intake, there was little evidence for effect modification of the association of prenatal air pollutant exposures and SRS scores (Table 3) or autism diagnosis (eTable 5; http://links.lww.com/EE/A285). Among mothers with an intake of vitamin B6 below the median, NO_2_ exposure was inversely associated with SRS score (−0.79 [−1.55, −0.45]; e.g., as NO_2_ increased, autism-related trait scores decreased, counter to hypothesis); among mothers with vitamin B6 intake above the median, NO_2_ exposure was positively associated with SRS score (0.32 [0.25, 1.53]). Although not statistically significant, and confidence intervals were wide, there was a trend for inverse associations of PM_2.5_ and NO_2_ with SRS score (decreases in autism-related traits with increasing exposure levels) and positive associations of O_3_ with SRS score (increases in autism-related traits with increasing exposure levels) among mothers with nutrient intakes below cutoffs. The direction of associations was mixed for mothers with nutrient intakes above cutoffs.

**Table 3.** The association of average prenatal exposure to air pollutants with SRS total raw score and autism diagnosis in the EARLI study, stratified by nutrient intake below versus above the median^a^

	SRS score			
	N below/N above cutoff	Below cutoff	Above cutoff	*P* for interaction^b^
		Regression coefficient (95% confidence interval)		
PM_2.5_				
Unstratified	126	−0.17 (−3.29, 0.91)		
Vitamin D (below vs. above RDA)	62/64	−1.27 (−3.45, 2.65)	−0.33 (−3.79, 2.70)	0.98
Folate	66/60	**−1.16 (−2.97, −0.41**)	−0.04 (−5.33, 3.16)	0.42
Vitamin B12	66/60	−3.22 (−7.31, 2.71)	1.01 (−0.77, 3.83)	**0.02**
Vitamin B6	69/57	−1.30 (−6.38, 0.15)	**2.69 (1.39, 4.96**)	0.54
Choline	63/63	1.48 (−2.60, 3.72)	−1.93 (−3.07, 0.37)	0.58
Betaine	63/63	−1.74 (−4.72, 3.08)	−0.64 (−1.80, 1.85)	0.30
Total omega 3s	67/59	−0.04 (−3.86, 1.17)	0.99 (−2.97, 4.43)	0.86
Total omega 6s	60/66	−0.44 (−2.06, 4.86)	−1.30 (−4.36, 2.57)	0.76
NO_2_				
Unstratified	126	−0.01 (−0.77, 0.83)		
Vitamin D (below vs. above RDA)	62/64	−0.28 (−1.00, 1.47)	−0.13 (−0.60, 1.60)	0.79
Folate	66/60	−0.45 (−0.94, 0.36)	0.36 (−0.31, 1.23)	0.55
Vitamin B12	66/60	−0.77 (−1.17, 1.28)	0.28 (−0.53, 1.12)	0.95
Vitamin B6	69/57	**−0.79 (−1.55, −0.45**)	**0.32 (0.25, 1.53**)	0.73
Choline	63/63	0.91 (−0.87, 1.83)	−0.23 (−0.93, 0.18)	**0.04**
Betaine	63/63	−0.28 (−1.63, 0.95)	−0.06 (−0.48, 0.63)	0.63
Total omega 3s	67/59	0.10 (−0.90, 0.86)	0.27 (−0.76, 0.92)	**0.003**
Total omega 6s	60/66	−0.16 (−1.21, 0.40)	0.08 (−0.72, 1.08)	0.85
O_3_				
Unstratified	126	0.35 (−0.51, 1.55)		
Vitamin D (below vs. above RDA)	62/64	0.66 (−0.81, 2.73)	0.34 (−1.75, 2.26)	0.77
Folate	66/60	0.18 (−1.22, 1.14)	1.10 (−0.51, 3.25)	0.78
Vitamin B12	66/60	0.75 (−2.03, 2.25)	0.68 (−0.74, 3.56)	0.28
Vitamin B6	69/57	0.40 (−1.20, 1.43)	0.00 (−0.89, 3.49)	0.68
Choline	63/63	1.60 (−0.83, 2.37)	0.13 (−0.65, 1.03)	0.99
Betaine	63/63	**0.64 (0.11, 1.98**)	−0.17 (−0.95, 1.69)	0.76
Total omega 3s	67/59	0.74 (−0.59, 2.30)	−0.29 (−1.36, 2.70)	0.74
Total omega 6s	60/66	0.22 (−1.33, 2.26)	0.72 (−0.65, 1.72)	0.76

Boldface considered statistically significant.

a Quantile regression models were performed to examine the association of air pollutants and SRS score at the 50th percentile. Estimates are per 1 unit increase in air pollutant exposures. Models were stratified by nutrient intake below and above the median (except vitamin D, which was below vs. above the RDA). Models were run separately for each air pollutant and nutrient. Models were adjusted for study site, maternal age, maternal race and ethnicity, maternal socioeconomic deprivation index, and child sex. Bolded values are considered statistically significant.

b *P* values are from adjusted, unstratified models testing the interaction term between each nutrient and air pollutant.

## Discussion

In this cohort study of 126 families with a prior child with an autism diagnosis, we did not find strong evidence for a moderating effect of prenatal nutrient intake on the association between air pollutant exposure and autism traits in a subsequent child. However, overall air pollutant exposures were relatively low, and nutrient intakes were at or above recommendations for most (but not all) participants in our study population. Despite null findings, this study builds on previous work and demonstrates the ability to examine the effects of prenatal exposure to a wide set of nutrients and environmental toxins in relationship to neurodevelopment using a Bayesian mixtures approach. Comparison to other study populations in future work is necessary to advance understanding of potential joint effects and interactions.

Previous studies, although limited in number, have generally supported the role of prenatal nutrient intake as a moderator of the relationship between environmental toxins and neurodevelopment, including autism.^18^ Intake of supplemental folic acid, in particular, has been shown to attenuate positive associations between prenatal exposure to pesticides, phthalates, and air pollutants and autism outcomes.^19,46,47^ Folic acid influences gene methylation and antioxidant pathways, the same pathways that are directly impacted by environmental toxins. For example, in rodent models, prenatal air pollutant exposure causes neuroinflammation and oxidative stress, but these are attenuated with intake of folic acid and other vitamins on the methylation pathway (vitamins B6 and B12).^48^ There is less evidence to date on the role of fatty acids or vitamin D for the attenuation of environmental effects on autism development. However, midpregnancy serum PUFA levels modified the association of mercury with psychomotor development at age of 20 months,^23^ and maternal fatty acid intake modified the association of the pesticide 1,1,1-trichloro-2,2-bis(*p*-chlorophenyl)ethane (DDT) with motor and memory development at 42–60 months^22^ but not Bayley Scales of Infant Development II scores at 1–30 months.^21^ Fatty acids are involved in inflammation and oxidation, along with cell membrane maintenance, and are particularly important for neurodevelopment during the third trimester of pregnancy.^49^ Maternal prenatal plasma vitamin D concentration attenuated the association between prenatal air pollutant exposures and Bayley Scales of Infant Development scores at age 14 months, although the interaction was not significant.^20^ Vitamin D is involved in gene expression and immune modulation and deserves further attention given its independent association with autism outcomes.^15^

While the majority of studies examining air pollution exposure and autism have reported associations, results across these studies, in terms of pollutant, time period, and outcome measurement under study, are highly variable, suggesting the influence of multiple factors. It is possible that the lack of effect shown here could be due to the sample’s relatively low exposure to air pollution and high intake of beneficial nutrients compared to US standards. Air pollution levels were below EPA annual standards, although these standards were not determined on the basis of autism risk, and associations with autism outcomes have been documented at levels similar to those in this study.^19,50^ Nutrient intakes were also relatively high in this population who already had one child with autism. Nearly all used prenatal vitamins during their pregnancy, and more than half used them in the first month of pregnancy. Previous work examining the role of supplemental folic acid for autism has often used a stratification point of 800 mcg folic acid (1333 mcg dietary folate equivalents);^19,46^ in this sample, 75% of mothers reported intake above this level, although we included intake from both diet and supplements in this analysis. Intakes of vitamin B12 and B6 were similarly high. However, intakes of other nutrients were far below recommendations. Choline, an essential nutrient required for neuronal development and fat transport,^51,52^ is not currently included in most prenatal vitamins. Less than 10% of pregnant individuals in this sample met the AI level for choline. Additionally, few met the requirements for omega 3 or omega 6 fatty acids (specifically, ALA and LA). PUFAs have been associated with neurodevelopment, including autism, in previous studies.^53,54^ Regardless of the potential for attenuation of effects of environmental toxins, improving prenatal intake of choline and fatty acids is crucial to support healthy neurodevelopment.

These results may also be due to the unique characteristics of our sample. Given that all the families included in this study already had a child with autism, they may represent a group with increased genetic susceptibility to autism development. Perhaps in this context, environmental factors play a weaker role in the etiology of autism, although prenatal vitamin use was a significant moderator of the association between phthalates and autism diagnosis in a similar familial autism cohort.^55^ Studies of gene–nutrient^35^ or gene–environment interactions may be useful for examining moderating effects. This population was also largely non-Hispanic White, educated, and owned a home; perhaps with these socioeconomic protective factors in place, factors such as air pollution and nutrition have reduced potential for neurodevelopmental effects.

Strengths of this study include the examination of a wide variety of nutrients and the novel use of statistical mixture methods. Previous work has generally focused on a single nutrient or small group of nutrients, and several studies have focused on supplemental intake rather than intake from diet and supplements. To our knowledge, none have included a suite of nutrients involved in methylation, such as choline, despite evidence from animal models that these nutrients attenuate environmental toxin effects on neurodevelopment.^48,56,57^ Examination of these nutrients and air pollutants within a single mixtures model extends previous mixtures work that has focused on dietary factors^26^ or environmental factors^25^ separately. Compared to other mixtures models such as weighted quantile sum regression,^58^ BKMR allows for the most flexible examination of the intricate interrelationships among nutrients and air pollutants, without assuming the same effect directionality for each exposure.

Limitations of this study include the potential for exposure and outcome measurement errors. Air pollutant exposure was estimated based on residential address information. Individual-level exposure information, such as that collected via personal sensors,^59^ would provide more precise data. Ozone estimates were calculated using different methods than the other air pollutants because estimates from the spatiotemporal ensemble model were not available for ozone at the time of analysis. Future analyses should use the same estimation methods across pollutants. Measurement of self-reported diet is known to incur both error and bias, and thus there is the potential for error in nutrient levels used here.^60,61^ However, we minimized these factors by using prospectively reported diet during pregnancy, using a validated food frequency questionnaire, and adjusting for total energy intake. Additionally, SRS scores were measured once at 36 months of age using the preschool-based form. While SRS scores have been shown to have high reproducibility and strong test–retest reliability, preschool SRS scores are on average slightly lower than scores at later ages and may be more variable due to the developmental nature of autism and related traits.^29^ Future work should consider using SRS scores at later ages to account for potential differences in measurement at young ages. Finally, an important limitation is the small and relatively homogenous sample; while we were adequately powered for moderate effect sizes in line with those observed in prior work, the power to detect more modest associations was limited and several associations could not be estimated with a high degree of precision. Future studies using similar methods to examine these interactions in a larger and more diverse sample are needed.

## Conclusion

In this analysis, we highlight the potential for the use of mixtures-based regression models to examine the complex interactions of dietary factors and air pollutant exposures for autism development. Although we found limited evidence for interactions in this small sample, the use of these methods in a larger and more diverse sample is warranted. Future studies may identify individually modifiable dietary factors that could attenuate the effects of air pollutants on neurodevelopment.

## Conflicts of interest statement

The authors declare that they have no conflicts of interest with regard to the content of this report.

## Acknowledgments

EARLI study: Craig Newschaffer, Pennsylvania State University; M. Daniele Fallin, Emory University; Irva Hertz-Picciotto, UC Davis; and Lisa A. Croen, Kaiser Permanente.

## Supplementary Material
