# Supplementary material for: Prenatal air pollution and children’s autism traits score: Examination of joint associations with maternal intake of vitamin D, methyl donors, and polyunsaturated fatty acids using mixture methods
Source: Environ Epidemiol. 2024 Jun 21;8(4):e316. doi: 10.1097/EE9.0000000000000316 (PMC11196080; doi:10.1097/EE9.0000000000000316)
Supplement: Supplementary file 1 [file ee9-8-e316-s001.pdf]

Supplemental Digital Content

Environmental Epidemiology

Prenatal air pollution and children's autism traits score: examination of joint associations with maternal intake of vitamin D, methyl donors, and polyunsaturated fatty acids using mixture methods

Megan G. Bragg et al

eTable 1. Characteristics of EARLI participants overall (n=239) compared to those included in the analysis (n= 126) of nutrient intake, air pollution exposure, and autism outcomes

eTable 2. Spearman's correlations among nutrients and air pollutants for participants of the EARLI study (n = 126)

eTable 3. Posterior inclusion probabilities (PIPs) for the association of prenatal nutrient intakes and air pollutant exposures with SRS score in the EARLI study, from Bayesian kernel machine regression models (n = 126)

eTable 4. Bivariate associations of prenatal nutrient intake and air pollution exposure with autism traits scores in the EARLI study (n = 126)

eTable 5. The association of average prenatal exposure to air pollutants with autism diagnosis in the EARLI study, stratified by nutrient intake below vs above the median

eFigure 1. Bayesian kernel machine regression (BKMR) results for the association of prenatal nutrient intake and air pollution exposures with child SRS total raw score in the EARLI study, with further adjustment for covariates (n = 126)

eFigure 2. Bayesian kernel machine regression (BKMR) results for the association of prenatal nutrient intake and trimester-specific air pollution exposures with child SRS total raw score in the EARLI study (n = 126)

eFigure 3. Bayesian kernel machine regression (BKMR) results for the association of prenatal nutrient intake in the second half of pregnancy and air pollution exposures with child SRS total raw score in the EARLI study (n = 79)

eFigure 4. Probit Bayesian kernel machine regression (BKMR) results for the association of prenatal nutrient intake and air pollution exposures with child Autism diagnosis in the EARLI study (n = 144)

eTable 1. Characteristics of EARLI participants overall (n=239) compared to those included in the analysis (n= 126) of nutrient intake, air pollution exposure, and autism outcomes

	All EARLI (n=239)	Included (n = 126)
	Median (IQR) or n (%)	
Child characteristics		
Female, n (%)	114 (47.7)	55 (43.7)
Missing	<5	0 (0.0)
SRS total raw score	29.0 (18.0, 43.0)	29.5 (18.0, 42.0)
Missing	68 (28.5)	0 (0.0)
Autism diagnosis, n (%)	41 (17.2)	29 (23.0)
Missing	45 (18.8)	<5
Maternal and familial characteristics		
Maternal age (y)	34.0(31.0, 37.0)	34.0 (31.0, 37.0)
Missing	<5	0 (0.0)
Maternal race/ ethnicity[b], n (%)		
Non-Hispanic White	128 (53.6)	77 (61.1)
Hispanic	40 (16.7)	24 (19.0)
Other	71 (29.7)	25 (19.8)
Parity (number of live children prior to study pregnancy), n (%)		
1	102 (43.7)	61 (48.4)
2	90 (37.7)	44 (34.9)
3 or more	41 (17.2)	20 (15.9)
Missing	6 (2.5)	<5
Used prenatal vitamin/supplement during first month of pregnancy, n (%)		
Yes	141 (59.0)	72 (57.1)
No	93 (38.9)	53 (42.1)
Missing	5 (2.1)	<5
Individual socioeconomic deprivation index	0.40 (0.1,0.6)	0.3 (0.1, 0.6)
Maternal education, n (%)		
High school or less	29 (12.1)	16 (12.7)
Some college or more	204 (85.4)	109 (86.5)
Missing	6 (2.5)	<5
Paternal education, n (%)		
High school or less	47 (19.7)	26 (20.6)
Some college or more	181 (75.7)	96 (76.2)
Missing	11 (4.6)	<5
Household income, n (%)		

<$50K	62 (25.9)	25 (19.8)
$50-100K	71 (29.7)	46 (36.5)
$100K+	95 (39.8)	51 (40.4)
Missing	11 (4.6)	<5
Marital status, n (%)		
Married	189 (79.1)	103 (81.7)
All other situations	44 (18.4)	22 (17.5)
Missing	6 (2.5)	<5
Home ownership, n (%)		
Owned with a mortgage	136 (56.9)	76 (60.3)
Owned free & clear	8 (3.4)	<5
Rented for cash (or occupied without rent)	90 (37.7)	45 (35.7)
Missing	6 (0.03)	<5
EARLI Site, n (%)		
Drexel (PA)	62 (26.0)	33 (26.2)
Johns Hopkins (MD)	55 (23.0)	30 (23.8)
Kaiser Permanente (CA)	69 (28.9)	46 (36.5)
UC Davis (CA)	53 (22.2)	17 (13.5)

EARLI - Early Autism Risk Longitudinal Investigation

[a] P values are from t-tests and Chi-square tests of the difference between included and excluded participants.

[b] Race and ethnicity were combined and collapsed due to small n's < 5 in cells.

eTable 2. Spearman's correlations among nutrients and air pollutants for participants of the EARLI study (n = 126)

	Vitamin D	Folate	Vitamin B12	Vitamin B6	Choline	Betaine	Total omega 3s	Total omega 6s	PM2.5	NO2	O3
Vitamin D (mcg/d)	1.00										
Folate (mcg DFE/d)	0.60	1.00									
Vitamin B12 (mcg/d)	0.66	0.54	1.00								
Vitamin B6 (mg/d)	0.50	0.66	0.70	1.00							
Choline (mg/d)[a]	0.17	0.01	0.07	0.03	1.00						
Betaine (mg/d)[a]	0.13	0.03	0.12	0.07	0.22	1.00					
Total omega 3s (g)	0.23	0.15	0.29	0.29	0.24	0.41	1.00				
Total omega 6s (g)[a]	0.11	0.13	0.17	0.21	0.16	0.25	0.49	1.00			
$PM_{2.5}$ ($\mu g/m^3$)	0.09	0.09	-0.03	0.04	-0.10	0.01	-0.001	-0.12	1.00		
NO_2 (ppb)	0.09	0.05	0.06	-0.06	-0.12	0.07	0.01	-0.12	0.47	1.00	
O_3 (ppb)	-0.04	0.11	0.003	0.13	0.01	0.07	0.18	0.07	0.03	-0.18	1.00

DFE – Dietary Folate Equivalents; EARLI - Early Autism Risk Longitudinal Investigation

[a] Does not include intake from supplements

eTable 3. Posterior inclusion probabilities (PIPs) for the association of prenatal nutrient intakes and air pollutant exposures with SRS score in the EARLI study, from Bayesian kernel machine regression models (n = 126)[a]

	Unadjusted			Adjusted		
	Individual PIP	Group PIP	Conditional PIP	Individual PIP	Group PIP	Conditional PIP
Nutrient						
Vitamin D (mcg/d)	0.09	0.20	1.00	0.07	0.19	1.00
Folate (mcg DFE/d)	0.09	0.32	0.17	0.04	0.28	0.15
Vitamin B12 (mcg/d)	0.05	0.32	0.11	0.06	0.28	0.12
Vitamin B6 (mg/d)	0.19	0.32	0.32	0.12	0.28	0.26
Choline (mg/d)	0.10	0.32	0.19	0.10	0.28	0.32
Betaine (mg/d)	0.09	0.32	0.20	0.06	0.28	0.14
Total omega 3s (g)	0.10	0.24	0.57	0.05	0.20	0.55
Total omega 6s (g)	0.08	0.24	0.43	0.06	0.20	0.45
Air pollutant						
$PM_{2.5}$ (μg/m^3)	0.13	0.31	0.35	0.05	0.31	0.23
NO_2 (ppb)	0.14	0.31	0.37	0.07	0.31	0.26
O_3 (ppb)[b]	0.08	0.31	0.28	0.12	0.31	0.51

DFE – Dietary Folate Equivalents; EARLI - Early Autism Risk Longitudinal Investigation; SRS – Social Responsiveness Scale

[a] PIPs are from Bayesian kernel machine regression analyses examining the association of prenatal nutrient intake and air pollution exposures with child SRS total raw score. PIPs describe the relative strength of the association of an exposure (or group of exposures) with SRS score. Individual PIPs describe the relative strength of associations with each unique exposure. Group PIPs describe the relative strength of the association with a group of exposures. Conditional PIPs describe the relative strength of the association with a unique exposure within a group. Adjusted models were adjusted for study site, maternal age, maternal race and ethnicity, maternal socioeconomic deprivation index, and child sex.

eTable 4. Bivariate associations of prenatal nutrient intake and air pollution exposure with autism traits scores in the EARLI study (n = 126)[a]

	SRS score	
	Unadjusted	Adjusted
	Regression coefficient (95% CI)	
Nutrient		
Vitamin D (mcg/d)	-0.01 (-0.11, 0.14)	0.04 (-0.06, 0.20)
Folate (mcg DFE/d)	-0.005 (-0.01, 0.004)	-0.001 (-0.01, 0.001)
Vitamin B12 (mcg/d)	-0.003 (-0.02, 0.03)	0.003 (-0.01, 0.03)
Vitamin B6 (mg/d)	-0.12 (-0.30, 0.01)	-0.07 (-0.28, 0.02)
Choline (mg/d) [b]	-0.01 (-0.07, 0.01)	-0.01 (-0.07, 0.01)
Betaine (mg/d) [b]	-0.08 (-0.15, 0.03)	0.01 (-0.03, 0.09)
Total omega 3s (g)	-3.58 (-12.92, 11.78)	-0.44 (-6.81, 3.21)
Total omega 6s (g) [b]	0.41 (-1.02, 1.65)	-0.65 (-0.99, 0.34)
Air pollutant		
$PM_{2.5}$ (µg/m³)	**-1.66 (-3.07, -0.19)**	-0.17 (-3.29, 0.91)
NO_2 (ppb)	-0.07 (-0.74, 0.45)	-0.01 (-0.77, 0.83)
O_3 (ppb)	-0.62 (-1.50, 0.28)	0.35 (-0.51, 1.55)

DFE – Dietary Folate Equivalents; EARLI - Early Autism Risk Longitudinal Investigation; SRS – Social Responsiveness Scale

[a] Quantile regression models were performed to examine the association of nutrient intake air pollutant exposures with SRS score at the 50th percentile. Estimates are per 1 unit increase in nutrient or air pollutant exposures. Models were run separately for each nutrient and air pollutant. Adjusted models were adjusted for study site, maternal age, maternal race and ethnicity, maternal socioeconomic deprivation index, and child sex.

[b] Does not include intake from supplements

eTable 5. The association of average prenatal exposure to air pollutants with autism diagnosis in the EARLI study, stratified by nutrient intake below vs above the median[a]

	N below / N above cutoff	Case N below / N above cutoff	Unadjusted		Adjusted		
			Below cutoff	Above cutoff	Below cutoff	Above cutoff	P for interaction[b]
			OR (95% CI)				
$PM_{2.5}$							
Unstratified	144	33	0.97 (0.79, 1.20)		1.12 (0.86, 1.50)		
Vitamin D (below vs above RDA)	68/76	13/20	1.12 (0.80, 1.66)	0.88 (0.66, 1.16)	1.22 (0.73, 2.22)	1.11 (0.78, 1.59)	0.80
Folate	71/73	16/17	1.00 (0.77, 1.32)	0.91 (0.64, 1.32)	1.20 (0.80, 1.86)	0.97 (0.61, 1.56)	0.69
Vitamin B12	73/71	16/17	0.98 (0.72, 1.36)	0.96 (0.72, 1.29)	0.97 (0.59, 1.59)	1.16 (0.81, 1.72)	0.35
Vitamin B6	72/72	18/15	0.97 (0.74, 1.31)	0.96 (0.71, 1.33)	1.10 (0.76, 1.61)	1.08 (0.68, 1.78)	0.53
Choline	73/71	18/15	0.95 (0.70, 1.28)	0.99 (0.74, 1.37)	1.26 (0.82, 2.0)	1.07 (0.73, 1.63)	0.22
Betaine	72/72	22/11	0.96 (0.74, 1.25)	1.02 (0.72, 1.54)	1.27 (0.90, 1.23)	1.02 (0.59, 1.97)	0.77
Total omega 3s	72/72	16/17	1.16 (0.84, 1.67)	0.84 (0.63, 1.12)	1.29 (0.84, 2.08)	0.97 (0.54, 1.74)	0.08
Total omega 6s	72/72	17/16	1.13 (0.82, 1.65)	0.86 (0.65, 1.15)	3.50 (1.58, 11.28)	0.81 (0.54, 1.19)	0.16
NO_2							
Unstratified	144	33	0.99 (0.92, 1.05)		1.0 (0.92, 1.08)		
Vitamin D (below vs above RDA)	68/76	13/20	0.99 (0.90, 1.10)	0.98 (0.90, 1.07)	0.94 (0.82, 1.07)	1.05 (0.93, 1.18)	0.35
Folate	71/73	16/17	0.96 (0.87, 1.04)	1.03 (0.93, 1.13)	0.97 (0.85, 1.11)	1.05 (0.93, 1.20)	0.05
Vitamin B12	73/71	16/17	0.96 (0.87, 1.05)	1.02 (0.93, 1.12)	0.94 (0.82, 1.08)	1.05 (0.94, 1.19)	0.98
Vitamin B6	72/72	18/15	0.91 (0.82, 1.00)	1.07 (0.97, 1.18)	0.95 (0.84, 1.07)	1.08 (0.95, 1.25)	0.71

Choline	73/71	18/15	0.95 (0.86, 1.05)	1.02 (0.93, 1.12)	0.98 (0.86, 1.12)	1.03 (0.92, 1.16)	0.57
Betaine	72/72	22/11	0.96 (0.88, 1.06)	1.02 (0.92, 1.14)	0.99 (0.89, 1.12)	1.01 (0.86, 1.19)	0.14
Total omega 3s	72/72	16/17	1.05 (0.94, 1.18)	0.95 (0.86, 1.03)	1.08 (0.95, 1.25)	0.95 (0.76, 1.16)	0.66
Total omega 6s	72/72	17/16	0.93 (0.84, 1.03)	1.03 (0.94, 1.13)	0.98 (0.85, 1.13)	1.02 (0.91, 1.15)	0.71
O_3							
Unstratified	144	33	1.01 (0.92, 1.10)		1.05 (0.93, 1.18)		
Vitamin D (below vs above RDA)	68/76	13/20	1.05 (0.91, 1.20)	0.98 (0.86, 1.11)	1.14 (0.94, 1.41)	1.0 (0.86, 1.19)	0.61
Folate	71/73	16/17	0.95 (0.83, 1.08)	1.07 (0.94, 1.21)	1.00 (0.82, 1.21)	1.04 (0.89, 1.23)	0.98
Vitamin B12	73/71	16/17	1.01 (0.89, 1.15)	1.00 (0.87, 1.14)	1.05 (0.85, 1.28)	1.05 (0.89, 1.24)	0.74
Vitamin B6	72/72	18/15	0.96 (0.84, 1.08)	1.06 (0.93, 1.22)	1.03 (0.86, 1.23)	1.04 (0.88, 1.24)	0.34
Choline	71/73	18/15	1.06 (0.94, 1.21)	0.94 (0.82, 1.08)	1.16 (0.96, 1.42)	0.97 (0.82, 1.15)	0.05
Betaine	72/72	22/11	1.00 (0.89, 1.11)	1.05 (0.89, 1.25)	1.00 (0.86, 1.15)	1.10 (0.85, 1.44)	0.97
Total omega 3s	72/72	16/17	1.07 (0.94, 1.23)	0.94 (0.82, 1.07)	1.05 (0.87, 1.27)	1.13 (0.89, 1.45)	0.84
Total omega 6s	72/72	17/16	1.08 (0.94, 1.24)	0.95 (0.83, 1.08)	1.22 (0.99, 1.52)	0.98 (0.82, 1.18)	0.09

EARLI - Early Autism Risk Longitudinal Investigation; RDA – Recommended Dietary Allowance

[a] Logistic regression models were performed to examine the association of air pollutants and autism diagnosis (yes/no). Models were stratified by nutrient intake below and above the median (except vitamin D, which was below vs above the RDA). Models were run separately for each air pollutant and nutrient. Adjusted models were adjusted for study site, maternal age, maternal race and ethnicity, maternal socioeconomic deprivation index, and child sex.

[b] P values are from adjusted, unstratified models testing the interaction term between each nutrient and air pollutant.

eFigure 1. Bayesian kernel machine regression (BKMR) results for the association of prenatal nutrient intake and air pollution exposures with child SRS total raw score in the EARLI study, with further adjustment for covariates (n = 126)

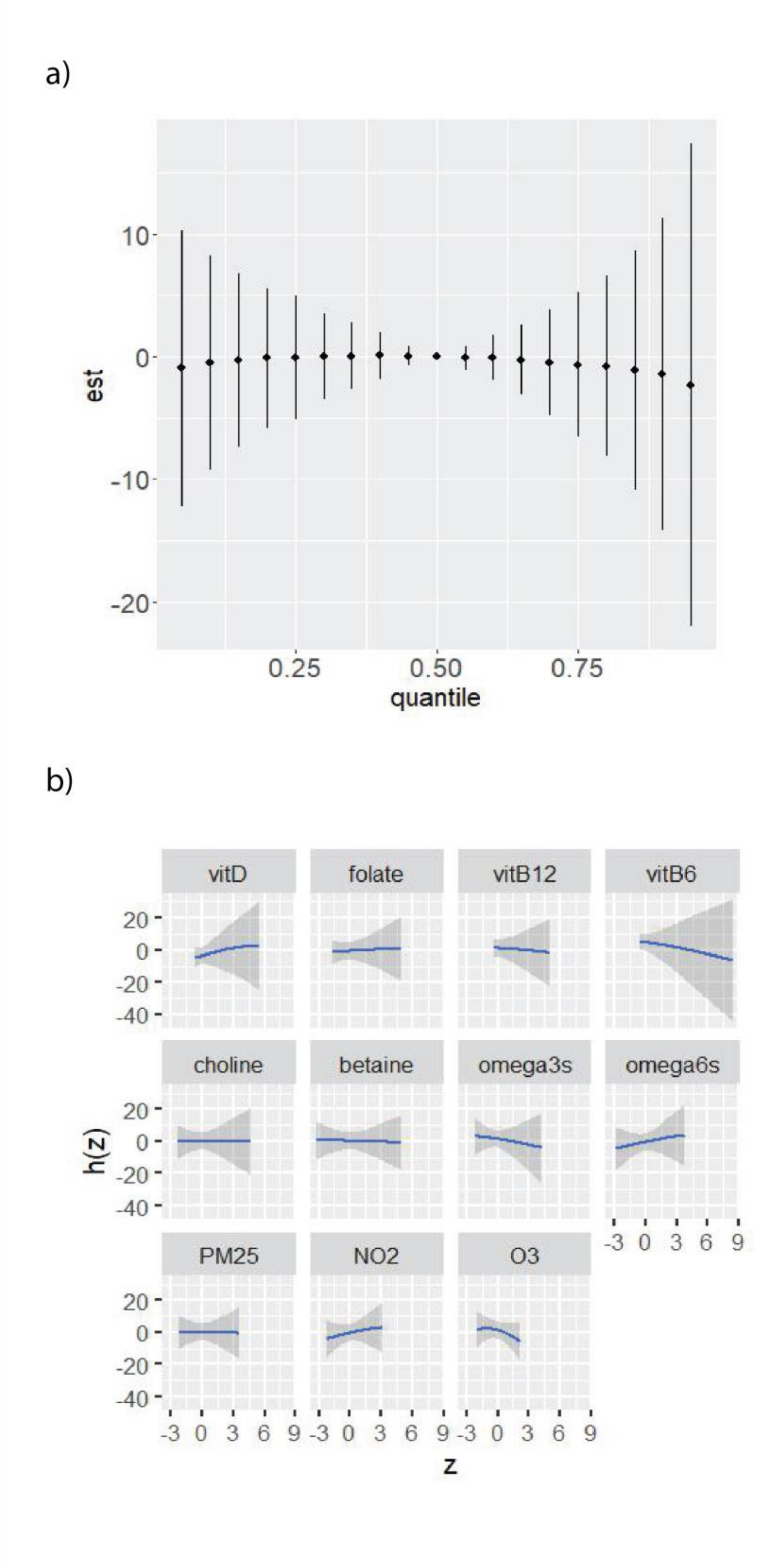

c)

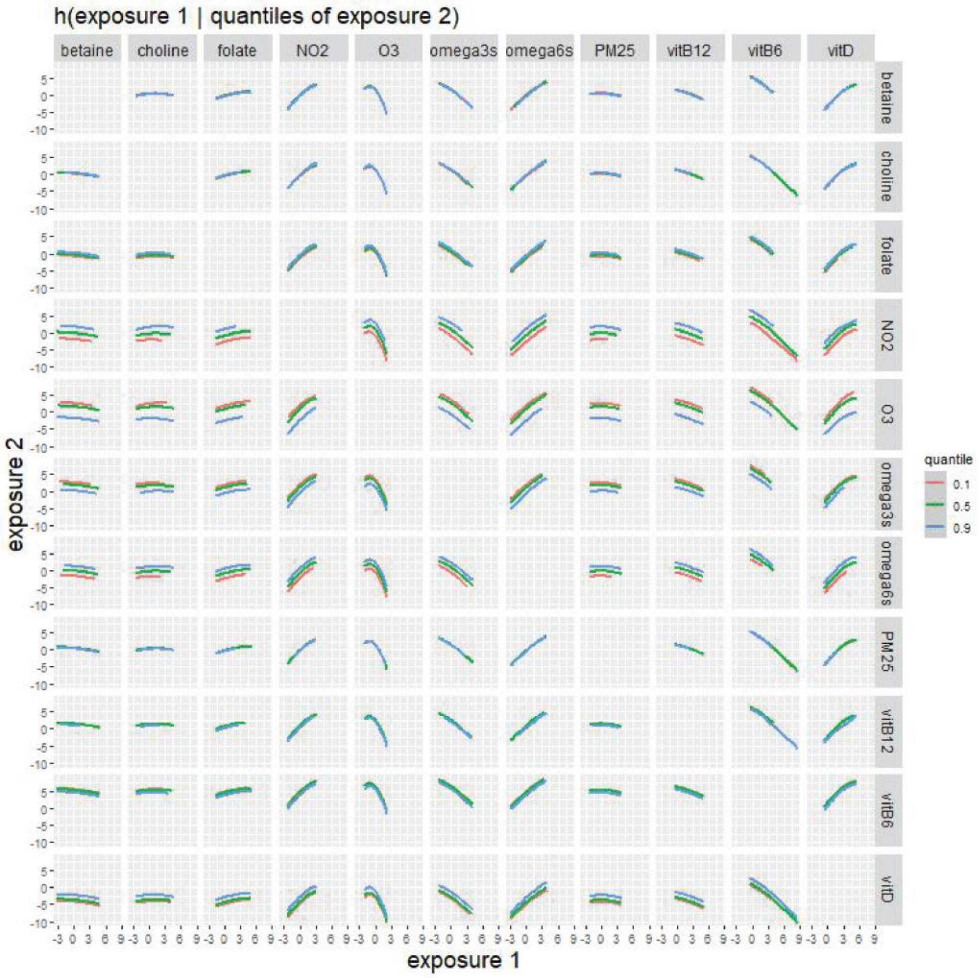
h(exposure 1 | quantiles of exposure 2)
betaine
choline
folate
NO2
O3
omega3s
omega6s
PM25
vitB12
vitB6
vitD
exposure 2
exposure 1
quantile
0.1
0.5
0.9

d)

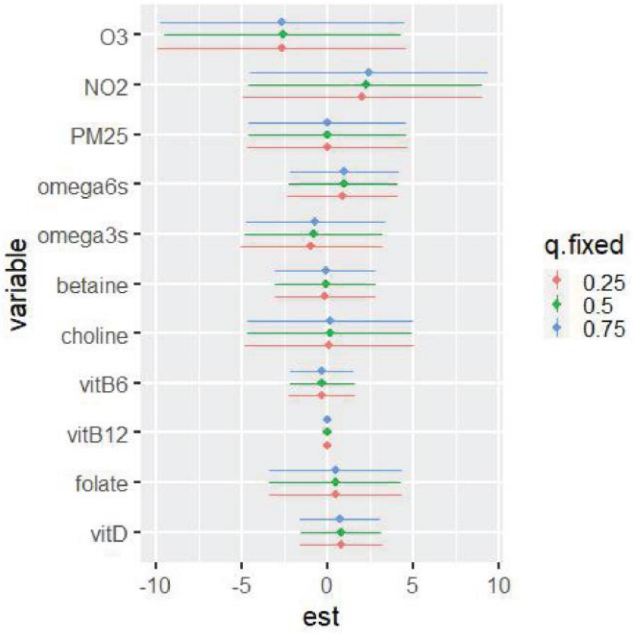
O3
NO2
PM25
omega6s
omega3s
betaine
choline
vitB6
vitB12
folate
vitD
variable
-10
-5
0
5
10
est
q.fixed
0.25
0.5
0.75

EARLI - Early Autism Risk Longitudinal Investigation; SRS – Social Responsiveness Scale

Results are adjusted for child sex, birth year and season, gestational age at birth, whether the child was ever breastfed; maternal age, race and ethnicity, socioeconomic deprivation index, prenatal vitamin/supplement use in first month of pregnancy, interpregnancy interval between end of most recent pregnancy and the current, maternal antidepressant use during pregnancy, pre-pregnancy BMI, gestational weight gain, parity, and Alternative Healthy Eating Index score for pregnancy (a measure of diet quality)[45], census tract-based socioeconomic status using the index of concentration at the extremes;[46] and study site. Air pollutant values are reverse coded. Plot A shows the association of the overall mixture with SRS score, when all exposures are set at the same quantile, compared to when all exposures are at their 50th percentile. Plot B shows the independent associations of each exposure with SRS, with all other exposures at their 50th percentile. Plot C shows the association of a single exposure with SRS score, when a second exposure is set at varying quantiles, illustrating potential bivariate interactive effects. Plot D shows the association of a single exposure with SRS score as it increases from its 25th to 75th percentile while all other exposures are set at specific quantiles. For all plots, covariates are held constant.

eFigure 2. Bayesian kernel machine regression (BKMR) results for the association of prenatal nutrient intake and trimester-specific air pollution exposures with child SRS total raw score in the EARLI study (n = 126)

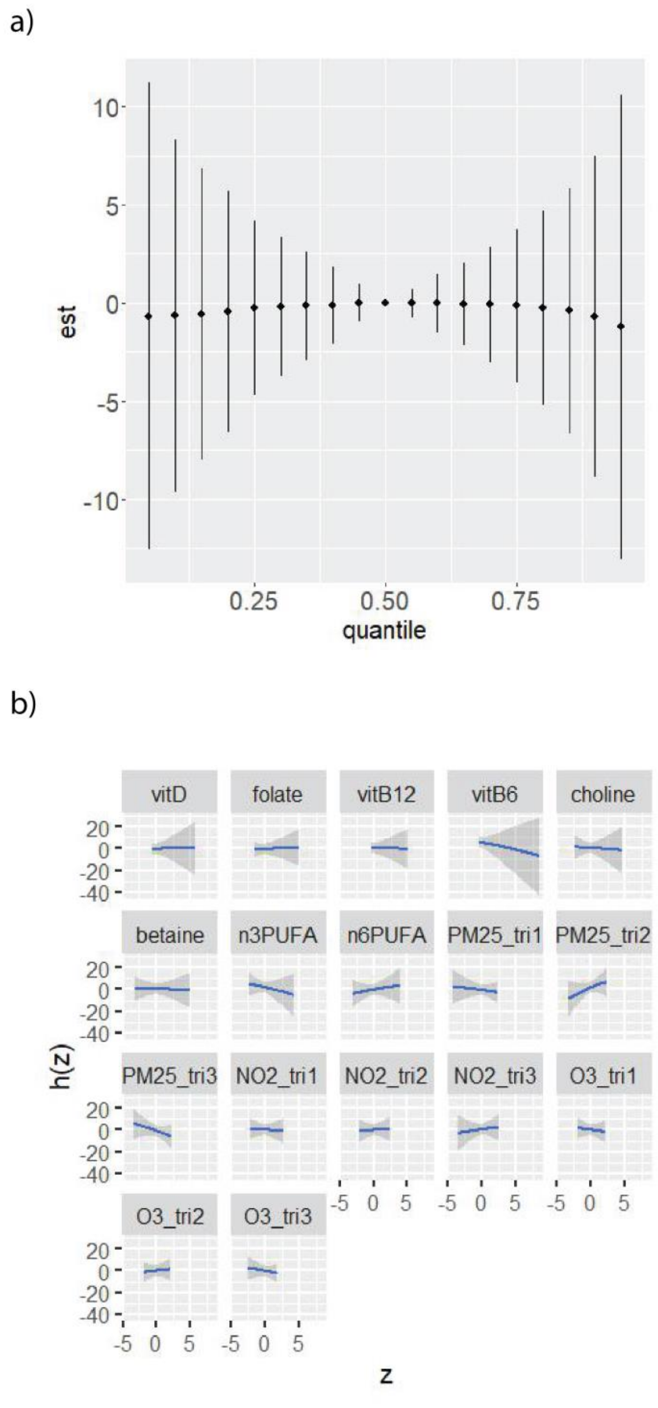

c)

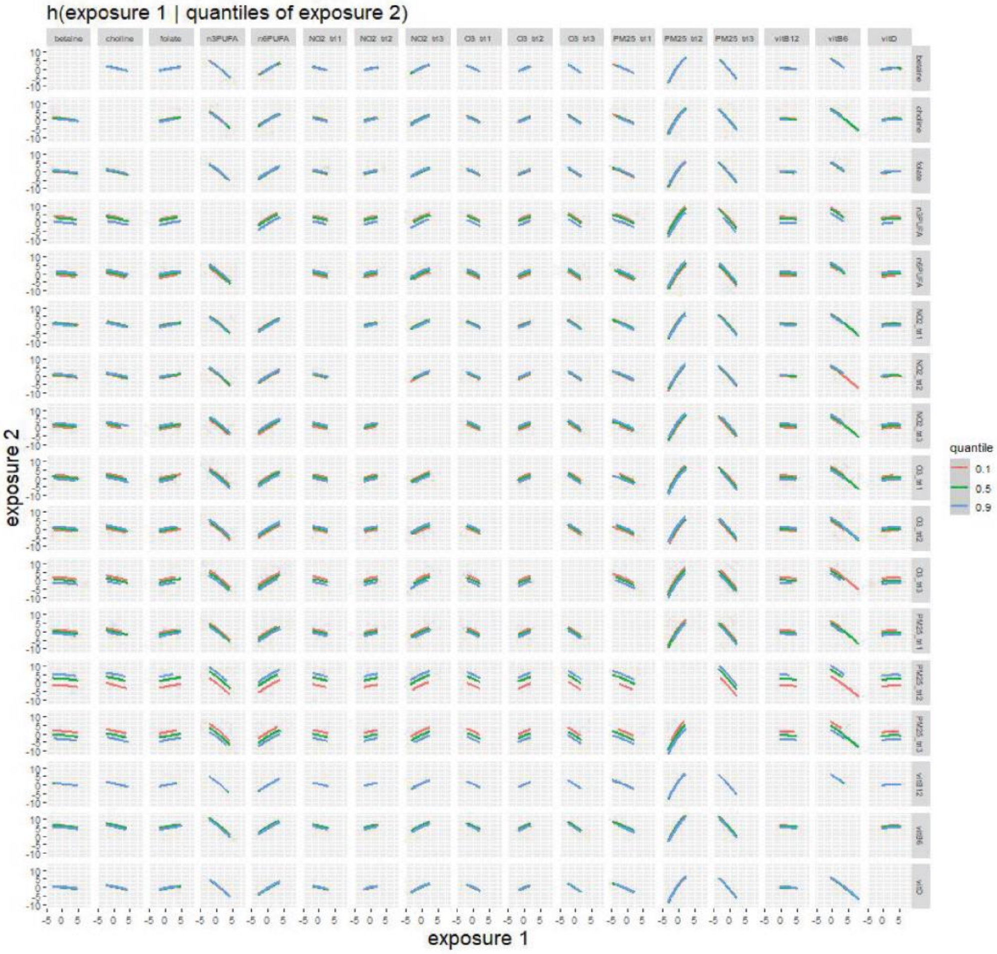
h(exposure 1 | quantiles of exposure 2)
betaine
choline
folate
n3PUFA
n6PUFA
NO2 tri1
NO2 tri2
NO2 tri3
O3 tri1
O3 tri2
O3 tri3
PM25 tri1
PM25 tri2
PM25 tri3
vitB12
vitB6
vitD
exposure 2
exposure 1
quantile
0.1
0.5
0.9

d)

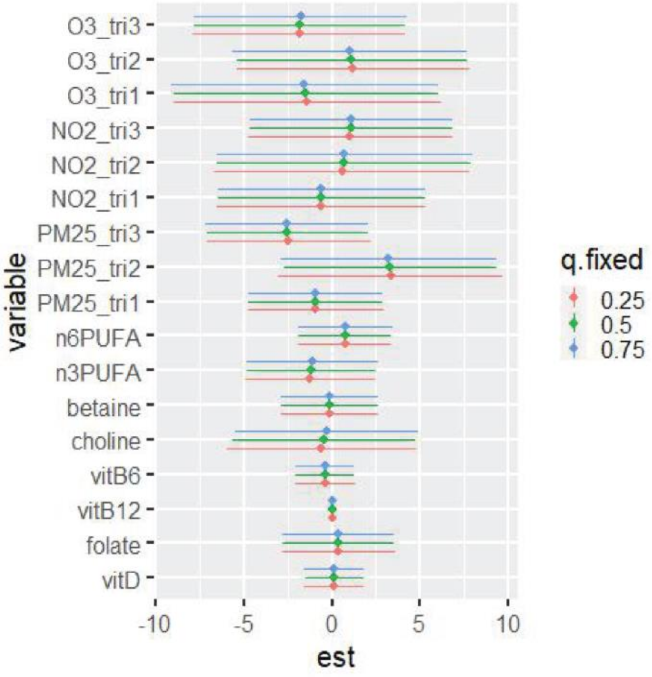
O3_tri3
O3_tri2
O3_tri1
NO2_tri3
NO2_tri2
NO2_tri1
PM25_tri3
PM25_tri2
PM25_tri1
n6PUFA
n3PUFA
betaine
choline
vitB6
vitB12
folate
vitD
variable
-10
-5
0
5
10
est
q.fixed
0.25
0.5
0.75

EARLI - Early Autism Risk Longitudinal Investigation; SRS – Social Responsiveness Scale

Results are adjusted for study site, maternal age, maternal race and ethnicity, maternal socioeconomic deprivation index, and child sex. Air pollutant values are reverse coded. Plot A shows the association of the overall mixture with SRS score, when all exposures are set at the same quantile, compared to when all exposures are at their 50th percentile. Plot B shows the independent associations of each exposure with SRS, with all other exposures at their 50th percentile. Plot C shows the association of a single exposure with SRS score, when a second exposure is set at varying quantiles, illustrating potential bivariate interactive effects. Plot D shows the association of a single exposure with SRS score as it increases from its 25th to 75th percentile while all other exposures are set at specific quantiles. For all plots, covariates are held constant.

eFigure 3. Bayesian kernel machine regression (BKMR) results for the association of prenatal nutrient intake in the second half of pregnancy and air pollution exposures with child SRS total raw score in the EARLI study (n = 79)

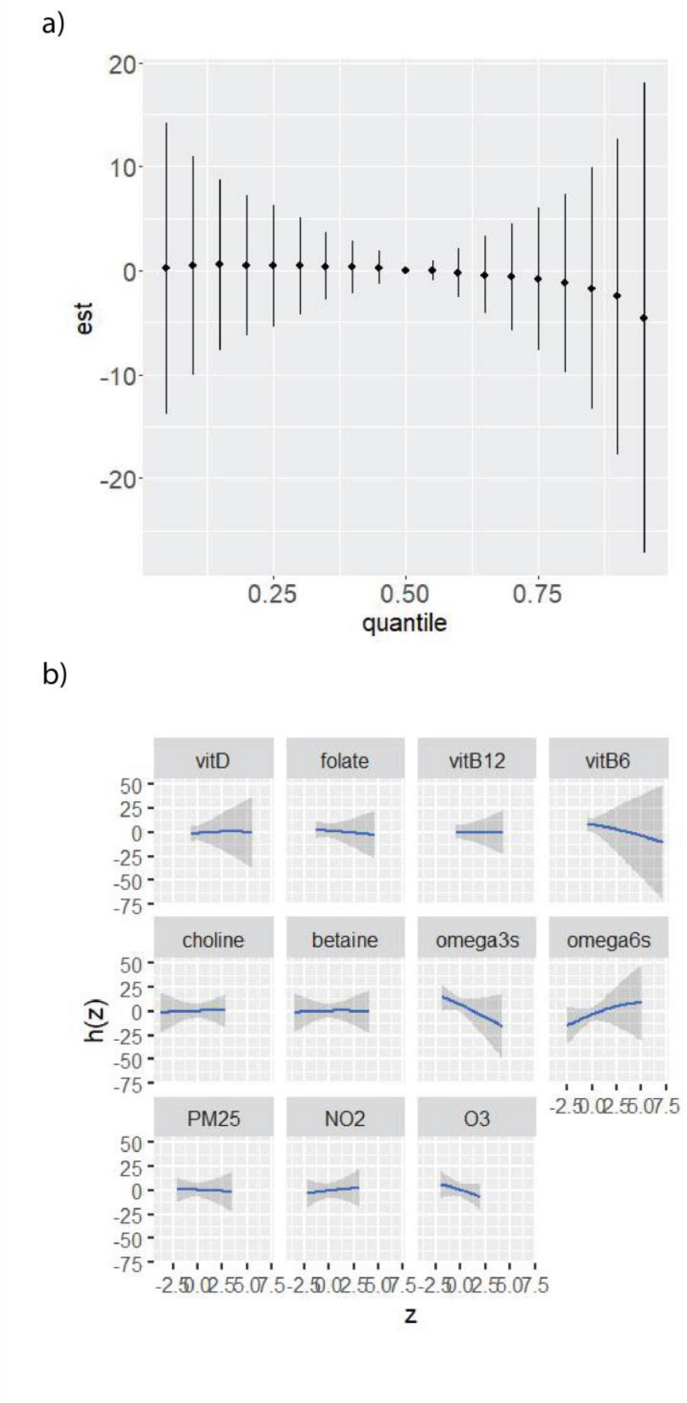

c)

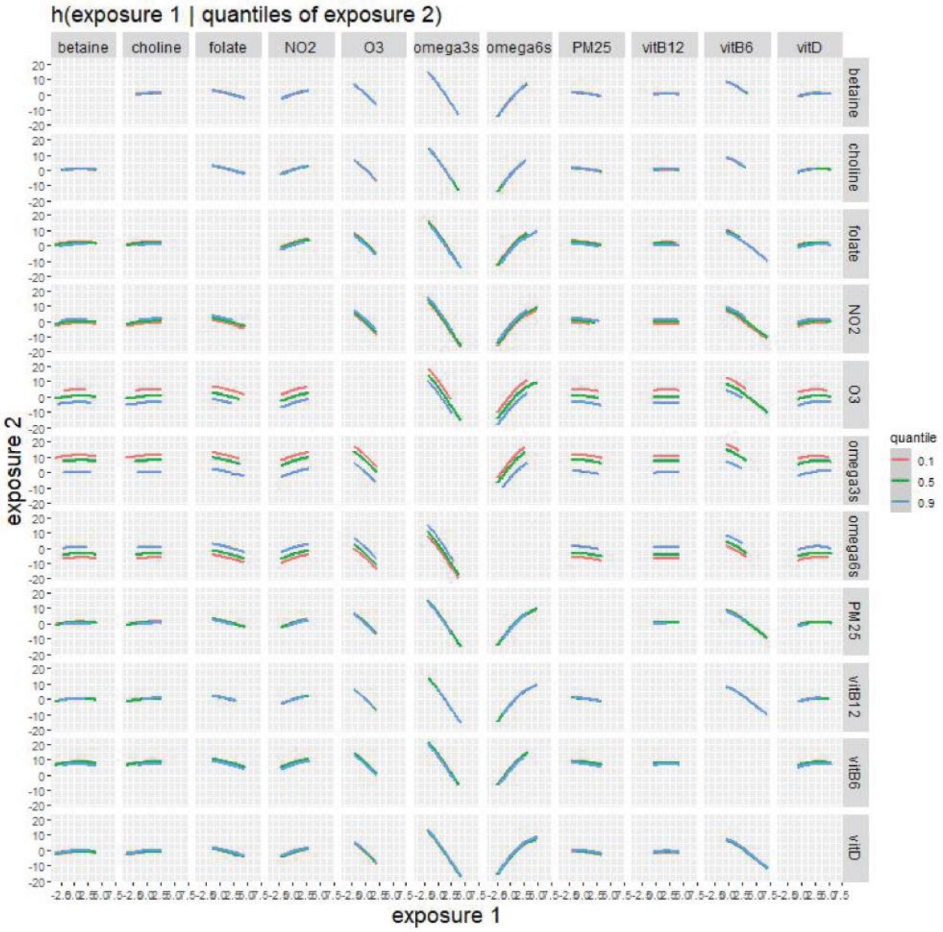

h(exposure 1 | quantiles of exposure 2)
betaine
choline
folate
NO2
O3
omega3s
omega6s
PM25
vitB12
vitB6
vitD
exposure 2
exposure 1
quantile
0.1
0.5
0.9

d)

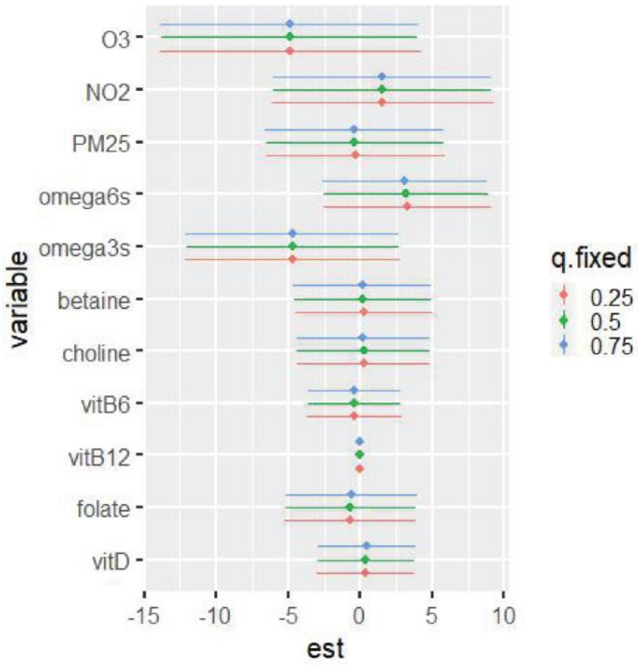

O3
NO2
PM25
omega6s
omega3s
betaine
choline
vitB6
vitB12
folate
vitD
variable
-15
-10
-5
0
5
10
est
q.fixed
0.25
0.5
0.75

EARLI - Early Autism Risk Longitudinal Investigation; SRS – Social Responsiveness Scale

Results are adjusted for study site, maternal age, maternal race and ethnicity, maternal socioeconomic deprivation index, and child sex. Air pollutant values are reverse coded. Plot A shows the association of the overall mixture with SRS score, when all exposures are set at the same quantile, compared to when all exposures are at their 50th percentile. Plot B shows the independent associations of each exposure with SRS, with all other exposures at their 50th percentile. Plot C shows the association of a single exposure with SRS score, when a second exposure is set at varying quantiles, illustrating potential bivariate interactive effects. Plot D shows the association of a single exposure with SRS score as it increases from its 25th to 75th percentile while all other exposures are set at specific quantiles. For all plots, covariates are held constant.

eFigure 4. Probit Bayesian kernel machine regression (BKMR) results for the association of prenatal nutrient intake and air pollution exposures with child autism diagnosis in the EARLI study (n = 144)

a)

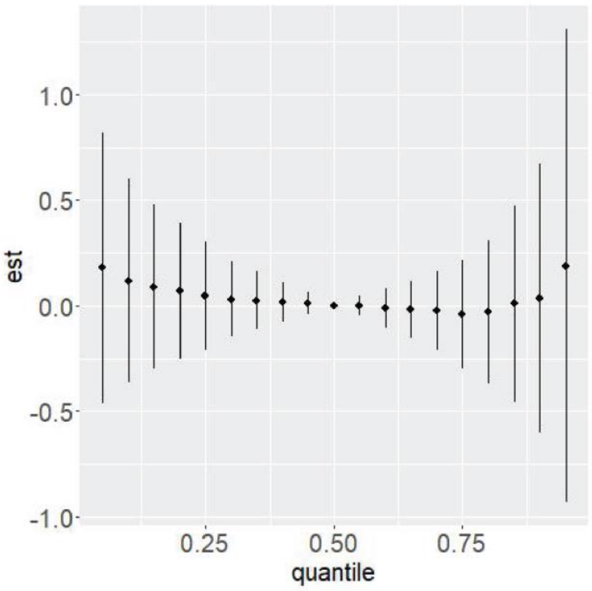

b)

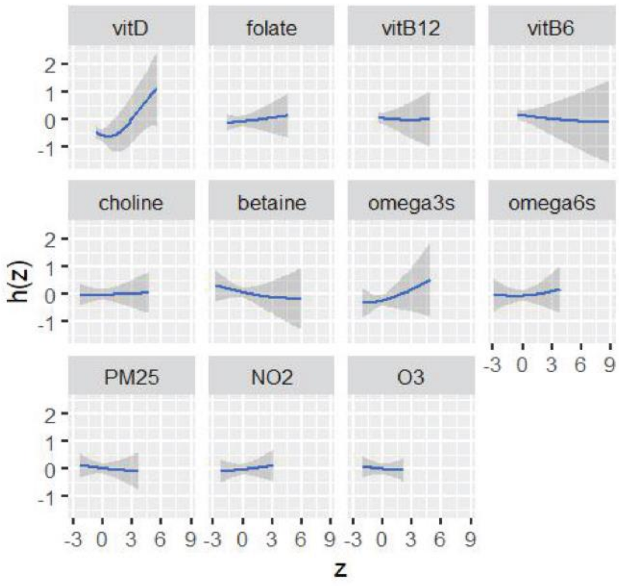

c)

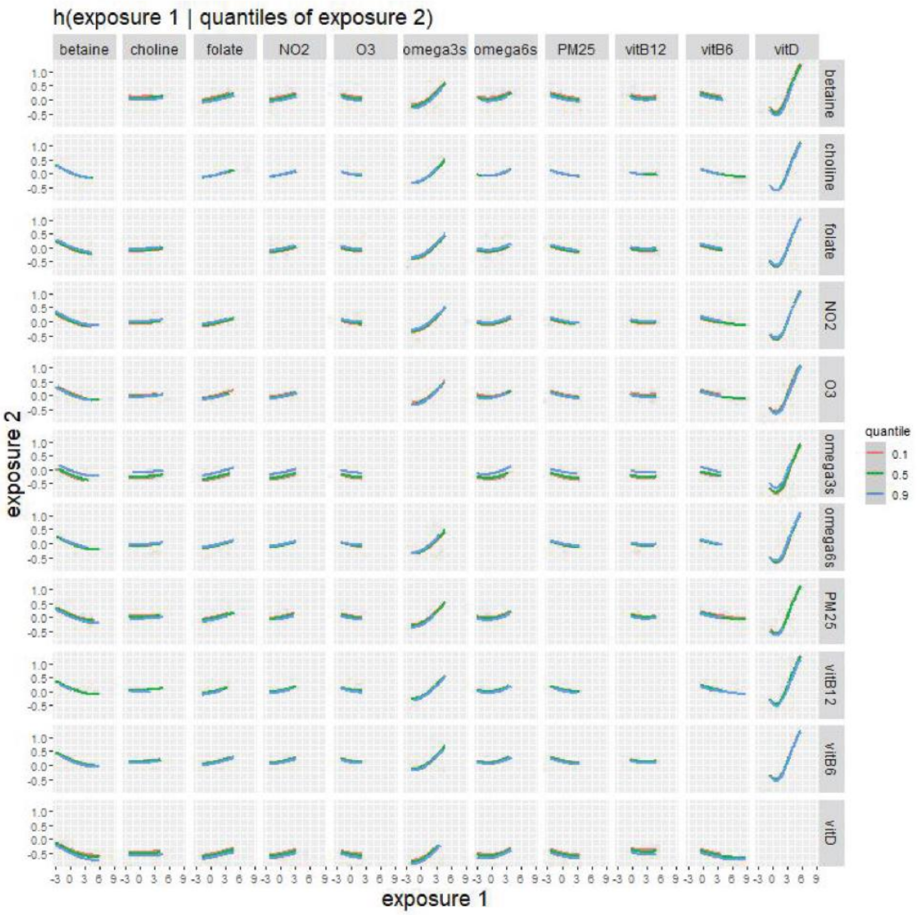
h(exposure 1 | quantiles of exposure 2)
betaine
choline
folate
NO2
O3
omega3s
omega6s
PM25
vitB12
vitB6
vitD
exposure 2
exposure 1
quantile
0.1
0.5
0.9

d)

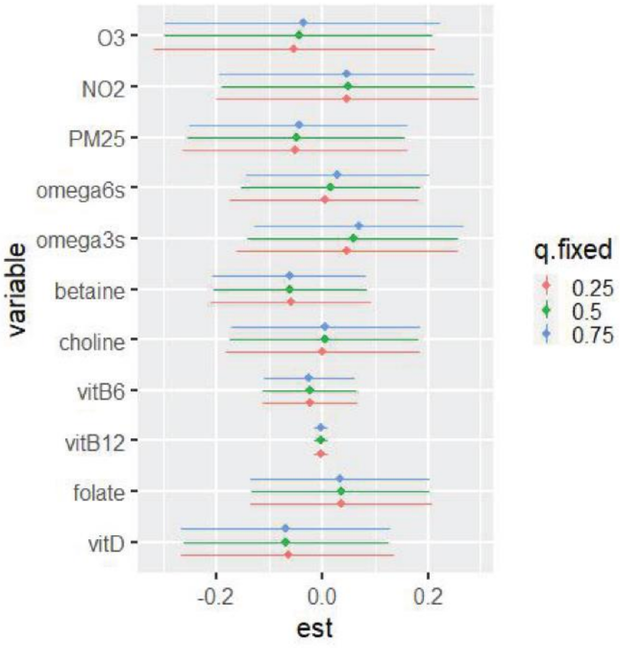
O3
NO2
PM25
omega6s
omega3s
betaine
choline
vitB6
vitB12
folate
vitD
variable
-0.2
0.0
0.2
est
q.fixed
0.25
0.5
0.75

EARLI - Early Autism Risk Longitudinal Investigation

Results are adjusted for study site, maternal age, maternal race and ethnicity, maternal socioeconomic deprivation index, and child sex. Air pollutant values are reverse coded. Plot A shows the association of the overall mixture with ASD status, when all exposures are set at the same quantile, compared to when all exposures are at their 50th percentile. Plot B shows the independent associations of each exposure with ASD status, with all other exposures at their 50th percentile. Plot C shows the association of a single exposure with ASD status, when a second exposure is set at varying quantiles, illustrating potential bivariate interactive effects. Plot D shows the association of a single exposure with ASD status as it increases from its 25th to 75th percentile while all other exposures are set at specific quantiles. For all plots, covariates are held constant.
